# Supplementary material for: Carers using assistive technology in dementia care at home: a mixed methods study
Source: BMC Geriatr. 2022 Jun 8;22:490. doi: 10.1186/s12877-022-03167-4 (PMC9173970; doi:10.1186/s12877-022-03167-4)
Supplement: Supplementary file 3 — Additional file 3. Carers’ Experience of Assistive Technology use in dementia – Explanatory interviews: Interview topic guide. [file 12877_2022_3167_MOESM3_ESM.pdf]

Additional file 3: Explanatory interviews - interview topic guide:

Pre-interview:

Participant to receive the information sheet and asked to read it through. Participant will be given a brief introduction to the research that includes a description of Assistive Technology.

- Introduce self
- Participant to be told what will happen during the interview process and reminded that the interview will also be audio recorded. Participant to be told that an anonymised transcript will be made from the audio recording. Participant to be reminded that they will remain anonymous, and that their data will be confidential.
- Participant given time to ask questions

Questions:

Background:
1. Can you tell me a bit about yourself and your current situation and living arrangements? – You have the option of not providing this information if you do not wish to.
2. How long have you been helping/looking after/caring for [person with dementia]? How did it come about?
3. How is your day-to-day life affected by helping, looking after, or caring for [person with dementia]?
4. Do you have health issues (physical and mental) that has an impact on your caring for [person with dementia]?
5. What support, if any, do you receive from other people? Does the [person with dementia] get formal/paid care?
6. What tasks that you help or support [person with dementia] with do you find challenging?

Use of Assistive Technology:
1. How do you and/or [person with dementia] use AT? Do you use AT with other family members?
2. Were there any ATs you could not obtain?
3. Do you think using this AT [list AT currently or most recently used] helps/helped you in caring for [person with dementia]?
4. If you have used an AT before and no longer are using it, what do you think prevented you or [person with dementia] from using it?
5. How happy are you with using this AT? Why do you say that?
6. Did you buy/use AT devices (when more than one) in any particular order, did that help you meet your/[person with dementia's] needs?

Impact of AT:
1. To what extent is AT integrated in your daily life/help/care that you provide for [person with dementia]?
2. How often do you use the AT?
3. When you consider all the help you are providing to [person with dementia], how does the AT help you? Do the AT cause any problems?
4. With which aspects of care has AT helped you?
5. What impact has the use of AT had on you and your quality of life or the burden experienced through your caring role? -

a. Manage physical effects – sleep, health, aches and pains?

b. Manage psychological effects – mood? Exhaustion, stress, anxiety, depression?

c. Emotionally – Spending more time with person with dementia, frustration, confidence?

d. Socially – friends, time for self, hobbies, vacation?

e. Manage employment or education (e.g. University) – Time away from work/study

f. With relationships – Strengthened caring for person with dementia, more time

g. Provide better choice and control in everyday life

Is there anything else you would like to add about your caregiving experience or AT?
